# Management of Orthostatic Hypotension: A Literature Review

**DOI:** 10.7759/cureus.3166

**Published:** 2018-08-20

**Authors:** Asad Ali, Nouman Safdar Ali, Neha Waqas, Chandur Bhan, Waleed Iftikhar, FNU Sapna, FNU Jitidhar, Abbas M Cheema, Malik Qistas Ahmad, Usama Nasir, Shahzad Ahmed Sami, Annum Zulfiqar, Asma Ahmed

**Affiliations:** 1 Medicine, CMH Lahore Medical College and Institute of Dentistry, Lahore, PAK; 2 Medicine, Jinnah Hospital/Allama Iqbal Medical College, Lahore, PAK; 3 Surgery, Shaikh Khalifa Bin Zayed Al Nahyan Medical & Dental College, Broken Bow, PAK; 4 Internal Medicine, Chandka Medical College Hospital, Larkana, PAK; 5 Internal Medicine, CMH Lahore Medical College and Institute of Dentistry, Lahore, PAK; 6 Internal Medicine, Burhani Hospital, Karachi, PAK; 7 Internal Medicine, Orthopedic and Medical Institute, Karachi, PAK; 8 Internal Medicine, Combined Military Hospital, Lahore, PAK; 9 Hematology-Oncology, University of Arizona Cancer Center, Tucson, USA; 10 Internal Medicine, CMH Medical and Dental College, Lahore, PAK; 11 Internal Medicine, Sheikh Zayed Medical College/Hospital, Lahore, PAK; 12 Fatima Jinnah Medical University, Punjab, PAK

**Keywords:** orthostatic hypotension, cardiology, blood pressure, baroreceptors, hypovolemia, cerebrovascular, syncope, geriatric, morbidity

## Abstract

In the older population, especially the hospitalized patients who are prone to dehydration and hypovolemia, orthostatic hypotension (OH) presents as a debilitating disease. How different pharmacological and non-pharmacological interventions affect the incapacitating symptoms (falls and episodes of syncope), morbidity, and mortality related to OH has become a topic of debate. OH can predispose to ischemic heart disease (IHD). A non-pharmacological approach consisting of mobilization, early lifestyle changes, and therapeutic maneuvers is the first choice in the management of these patients. Individuals with persistent symptoms require pharmaceutical therapy to increase blood volume and peripheral vascular resistance. This article summarizes the management of OH that is vital to cope with the needs of the growing geriatric populations.

## Introduction and background

Orthostatic hypotension (OH) is characterized by a sustained decrease in blood pressure (BP) on standing. This drop in BP must be more than 20 mmHg for systolic pressure or more than 10 mmHg for diastolic pressure three minutes after standing upright or during a head-up tilt at 60 degrees [1]. The greatest impact of OH is noticed within the elderly population [2]. Its prevalence among elderly nursing home residents is about 50% [3]. Among hospitalized patients, about 60% adults experience OH. In the setting of an acute illness, immobilization and polypharmacy in hospitals are risk factors for OH and its associated complications [3]. OH is usually associated with other entities such as hypertension and chronic kidney disease (CKD). Cognitive impairment increases morbidity and mortality and represents an independent risk factor for the appearance of cardiovascular and cerebrovascular diseases. Therefore, it accentuates the risk of falling, disability, and deterioration of the quality of life [4]. Studies show the relationship of OH with cognitive impairment, increasing age, and female sex [5-7].

Symptoms

OH may be asymptomatic in most cases with an unscathed cerebral autoregulation. However, these patients are also susceptible to falls and syncope [4]. Symptomatic patients usually suffer from dizziness, headache, vertigo, nausea, weakness, angina, syncope, and strokes. Elderly patients may suffer from alterations in cognition, slurred speech, visual disturbances, and falls with the previously stated symptoms [8]. Fatigue, exercise, alcohol, or a copious meal may aggravate the symptoms. OH symptoms affect the quality of life and increase the risk of myocardial infarction (MI) and death [9-10].

Diagnosis

There are no recommendations that prefer a specific method while diagnosing OH in the hospital setting, and thus, both head-up tilt-table testing and active standing approaches can be used [3]. OH assessment in patients includes measurement of BP and heart rate after lying supine for five minutes and after standing upright for one to three minutes [11]. Positive head-up tilt-table results demonstrate the above-described changes if the head-up tilt position is kept for three minutes, at an angle of a minimum of 60 degrees [8]. Establishing the diagnosis of OH may require monitoring BP for several days. For this purpose, it is necessary to record the vital signs of these patients at different times of the day and after certain possible triggers such as meals, medications, and exercise. Due to nocturnal natriuresis, the symptoms of hypotension tend to be more frequent in the early hours of the morning. It is recommended to measure BP at this time to obtain a more sensitive measure for the purpose of diagnosis [4].

The correct diagnosis of OH also requires a medical practitioner to take an exhaustive clinical history that includes comorbidities, medication compliance, current symptoms, detailed physical examination with BP measurements, electrocardiogram, general laboratory tests (complete hematology and glycemic and metabolic profiles with the objective to rule out metabolic, renal, or hematologic causes) [12].

Pathophysiology

An upright posture leads to pooling of 500-1000 ml of blood in the lower extremities and splanchnic and pulmonary circulations [12]. This leads to a drop in the systolic and diastolic pressures, with the reduction of venous return to the heart, decreased ventricular filling, and transient descent of the afterload. It causes the activation of compensatory mechanisms that include the activation of the sympathetic nervous system with the subsequent deactivation of the parasympathetic nervous system, the activation of baroreceptors in the carotid sinus, aortic arch, heart, and lungs, and finally, the activation of the renin-angiotensin-aldosterone system (RAAS), contributing to peripheral vasoconstriction. There is a dysregulation of these compensatory mechanisms, which leads to OH.

Considering the onset, OH is classified into two groups: acute OH and chronic OH. OH can be triggered by acute conditions such as valvular heart disease, hypovolemia, and acute illness [13]. Some drugs can also cause OH, including antipsychotics, diuretics, alpha and beta receptor blockers, tricyclic antidepressants, narcotics, sedatives, vasodilators, antiparkinsonian medications, and illicit drugs such as marijuana.

Chronic OH is caused by an autonomic dysfunction associated with the aging process. Chronic disorders that increase the risk of chronic OH include diabetes, Parkinson's disease, and multiple system atrophy [14]. Supine hypertension is also considered a risk factor. It is now known that plasma renin, angiotensin, and an inadequate response to mineralocorticoid receptor play a role as contributing factors. OH is rarely associated with primary neurodegenerative diseases such as Lewy body dementia, multiple system atrophy, and pure autonomic failure.

## Review

Management

The patient should register the symptoms and vital signs daily to assess the effectiveness of the applied treatment, the final objective of which is to improve the symptomatology and reestablish their independence in daily activities. In addition, the severity of OH coupled with the presence of comorbidities should be considered for the individualization of the treatment plan. In this sense, the treatment must be managed as a process of hierarchical steps that include both non-pharmacological and pharmacological approaches [4,15].

Nonpharmacological Treatment

The non-pharmacological approach is considered the first-line treatment and includes physical counter-maneuvers and lifestyle modifications. Initially, acute OH management starts with outlining the acute medical conditions (pain, sepsis, volume depletion, acute heart failure, stroke, cardiac arrhythmia, or anemia), ensuring that the patients fully understand the nature of OH and the possible clinical outcomes such as falls, syncope, and presyncope, and discussing the importance of lifestyle modifications [16].

External compression, through compression garments for abdominal and lower extremities, allows reduction of venous stasis and thereby orthostatic arterial hypotension, especially in the elderly patients. At their maximum efficiency, these external compression garments are able to handle pressures ranging between 15 and 40 mmHg [17]. To treat and prevent OH in hospitalized older patients, early mobilization and avoidance of physical deconditioning are recommended [18]. In these patients, simple positional changes play a vital role in improving OH without medications.

Physical counter-maneuvers, such as lunges, half-squats, and calf-raises, involve the contraction of large muscles (quadriceps and gluteals), causing an increase in venous return and, hence, decreasing OH symptoms [17]. In the same context, breathing-related counter-maneuvers have the ability to improve the venous return to the heart from the abdomen and the upper extremities, thus promoting the cardiovascular stability in patients suffering from OH [4].

Increase in the intravascular volume and avoidance of hypovolemia should be the final objectives of any treatment for OH to ensure the effectiveness of the pharmacological approach. Hence, an adequate intake of liquid (2.0-2.5 L/day) and a liberal salt diet (daily intake 10-20 g) is recommended. In addition, patients can consider the rapid ingestion of 500 ml of water (in about five minutes) for the rapid rise in systolic arterial pressure of 30 mmHg [17]. Diuretic use should be stopped or reduced in patients with OH secondary to hypovolemia. If diuretics are necessary, drugs causing OH, including nitrates, tricyclic antidepressants, diuretics, neuroleptics, and alpha blockers, should be stopped. For patients with an autonomic failure, raising the head of the bed by five to ten degrees (15 cm) at night decreases the pressure for diuresis and natriuresis through the reduction of renal perfusion [19]. With respect to lifestyle modifications, patients should avoid activities that increase the core body temperature and induce peripheral vasodilatation (such as utilization of hot tubs, saunas, or spas, prolonged hot showers, and excessive high intense exercise). It is also recommended to avoid alcohol intake. Ingestion of small frequent meals to avoid postprandial hypotension [4] with vitamins and iron supplementation is also beneficial [15].

The non-pharmacological treatment of OH is usually sufficient in mild diseases; however, pharmacological approaches should be considered in moderate and severe diseases [12].

Pharmacological Interventions

Pharmacological therapy focuses on either increasing blood volume or raising peripheral vascular resistance.

1. Increasing blood volume

Increasing blood volume is considered as a long-term strategy that depends on an increase in the baseline BP throughout the day [16]. Fludrocortisone increases the reabsorption of water and renal sodium. It also increases the sensitivity of a-adrenoreceptors to circulating catecholamines. The indicated initial dose of fludrocortisone is 0.1 to 0.2 mg daily early in the morning [20-21]. It can be increased gradually up to 1 mg per day.

2. Increasing peripheral vascular resistance

Using direct and indirect sympathomimetic agents, such as short-acting peripheral-selective alpha-1 adrenergic agonist medications, a higher level of peripheral vascular resistance can be achieved. This approach is applied in the case of patients with chronic OH and persistent symptoms regardless of volume expansion. 

Midodrine causes direct activation of the a-adrenoreceptors by desglymidodrine (active metabolite of midodrine), which reduces the venous pooling in the splanchnic circulation and the legs, increases the peripheral vascular resistance, and improves orthostatic BP [22]. The indicated dose of midodrine is 2.5 to 15 mg, once to thrice per day [23]. Considering the drug interactions, midodrine can interact with sympathomimetic or sympatholytic agents enhancing or decreasing the vasopressor action, respectively. With beta blockers or calcium channel blockers, midodrine can accentuate bradycardia, arrhythmias, or atrioventricular (AV) block, and with salt-retaining steroids, it can augment the hypertensive effect [12].

Droxidopa is a synthetic norepinephrine prodrug that is converted into norepinephrine by aromatic L-amino acid (DOPA) decarboxylase [24] in both central nervous system and peripheral tissues, thereby inducing peripheral arterial and venous vasoconstriction. It is indicated in neurogenic OH.

Prostaglandin inhibitors, such as nonsteroidal anti-inflammatory drugs (NSAIDs), which block the vasodilating effects of prostaglandins can increase BP, but their use should be limited due to their side effects in elderly patients, such as gastrointestinal bleeding, renal failure, and electrolyte abnormalities [23].

The alpha-2 adrenergic agonists, such as clonidine and yohimbine, can be used to treat OH, especially in Parkinson’s disease. For OH patients with autonomic dysfunction, clonidine activates peripheral alpha-2 adrenoreceptors, thereby increasing BP. However, given the existence of studies with controversial evidence about its mechanism of action, it should be used carefully in patients with OH [25-26]. Yohimbine is a central alpha-2 adrenergic antagonist that can increase the central sympathetic outflow in some patients with a residual sympathetic nervous system efferent output [27]. Another potential medication for the management of OH is pyridostigmine, an acetylcholinesterase inhibitor that improves sympathetic ganglion neurotransmission by increasing cholinergic signals. Pyridostigmine alone and with concurrent midodrine have shown the same findings [28]. Due to the discrete effect of pyridostigmine on BP, its exclusive use in mild disease has been recommended [28-29]. The recommended dose of pyridostigmine is 30-60 mg once to thrice per day. Adverse effects include diarrhea, constipation, anhidrosis, abdominal cramps, sialorrhea, urinary incontinence, and excessive sweating.

The ideal treatment is managed on an individual basis by progressively titrating its dose until it reaches the maximum tolerable levels. If symptoms persist, it has been suggested that a change or an addition of another pharmacological agent to the regimen is warranted, again titrating from a lower indicated initial dose to a higher one [15].

## Conclusions

As the healthcare settings in the world improve and the average human lifespan gradually rises, geriatric populations and age-related inevitable complications are on the rise. OH is a similar disorder related to aging and despite the existence of standard protocols for the management of OH, there is massive room for research. More in-depth studies to find out effective ways of managing this condition are warranted to improve the quality of life in our increasing elderly populations.
